# Dam mutants provide improved sensitivity and spatial resolution for profiling transcription factor binding

**DOI:** 10.1186/s13072-019-0273-x

**Published:** 2019-06-13

**Authors:** Tomasz Szczesnik, Joshua W. K. Ho, Richard Sherwood

**Affiliations:** 1 Victor Chang Cardiac Research Institute, Darlinghurst, NSW 2010 Australia; 2 St Vincent’s Clinical School, University of New South Wales, Darlinghurst, NSW 2010 Australia; 3 School of Biomedical Sciences, Li Ka Shing Faculty of Medicine, The University of Hong Kong, Pokfulam, Hong Kong, SAR China; 4 Division of Genetics, Department of Medicine, Brigham and Women’s Hospital, Harvard Medical School, Boston, MA 02115 USA; 5 Hubrecht Institute, 3584 CT Utrecht, The Netherlands

**Keywords:** Dam, DamID, Tcf7l2, Transcription factor

## Abstract

DamID, in which a protein of interest is fused to Dam methylase, enables mapping of protein-DNA binding through readout of adenine methylation in genomic DNA. DamID offers a compelling alternative to chromatin immunoprecipitation sequencing (ChIP-Seq), particularly in cases where cell number or antibody availability is limiting. This comes at a cost, however, of high non-specific signal and a lowered spatial resolution of several kb, limiting its application to transcription factor-DNA binding. Here we show that mutations in Dam, when fused to the transcription factor Tcf7l2, greatly reduce non-specific methylation. Combined with a simplified DamID sequencing protocol, we find that these Dam mutants allow for accurate detection of transcription factor binding at a sensitivity and spatial resolution closely matching that seen in ChIP-seq.

**Electronic supplementary material:**

The online version of this article (10.1186/s13072-019-0273-x) contains supplementary material, which is available to authorized users.

## Introduction

DamID is an enzymatic assay for detecting the location of protein-DNA interactions across the genome [1]. This technique uses the bacterial enzyme DNA adenine methyltransferase (Dam), which methylates the adenine within a sequence of G–A–T–C. In *E. coli*, methylation by Dam marks the original genome, directing mismatch repair to newly synthesised copies instead of the original and provides a layer of transcriptional control [2]. DamID takes advantage of the absence of any detectable adenine methylation, or functional consequences thereof, in mammals (for evidence in other eukaryotes see [3–5]) to repurpose it into marking sites of protein-DNA interactions. Dam is tethered to a protein of interest such that wherever it binds any nearby GATCs will be methylated [6]. Since methylation is a stable, covalent modification, it persists throughout DNA extraction and can be detected anytime afterwards by cleavage with adenine methylation-specific restriction enzymes: DpnI cleaves any methylated GATC and DpnII cleaves unmethylated GATCs. The protocol is completed by ligation of an adapter onto these cleaved methylation sites, amplification, and identification by nextgen sequencing (NGS) or hybridisation [7].

A major use of DamID is to profile protein-DNA binding under conditions unsuitable for the more ubiquitously used chromatin immunoprecipitation (ChIP). DamID’s use of a restriction digest followed by ligation and amplification in the absence of any lossy wash steps means it requires less starting material: a few thousand cells suffice instead of the many millions required for ChIP-seq [8] and otherwise results in a quicker and more straightforward protocol. Detection of methylation, however, is limited by the presence of GATCs which occur on average at 2.6 sites every kb in the mouse genome. Similarly, DamID’s use of fusion proteins avoids the need for antibodies, which are expensive to make, not available for many proteins, and are often non-specific. (This is of interest since closely related transcription factor often bind to different locations.) The downside of requiring fusion proteins is that these are typically expressed ectopically, which can lead to aberrant protein-DNA binding due to abnormally low or high expression and limits applicability to cases where transgenic cell lines or animals can be made. Thus, DamID is a compelling alternative to ChIP-seq, especially in cases where cell number or antibody availability is limiting.

Despite these benefits, DamID has seen limited usage. This is due to its substantial drawback of high background noise and low spatial resolution, likely stemming from Dam’s high enzymatic activity. In *E. coli*, Dam methylates most of the genome despite being expressed low [9]. Even when fused with a DNA-binding protein, Dam still methylates many off-target sites throughout the genome, and if expressed long or high enough it will completely saturate the genome with methylation [1]. The high methylation rate has made it necessary to use very low expression of the Dam fusion protein, most commonly with a leaky uninduced heat shock promoter [7] or more recently through translation reinitiation [8, 10]. Greater control over the expression of Dam constructs has also been achieved using inducible systems, allowing expression within specific cells and avoiding the toxicity from high methylation in whole organisms [11].

Even at low expression levels, there is still substantial off-target methylation resulting in a high correlation with unfused Dam [10, 12]. The usual solution is to subtract the methylation pattern of the unfused Dam control [7]. Any interaction effects are ignored by this strategy: processivity, competition between Dam and protein binding, and different diffusion/methylation rates of unfused Dam could all skew this normalisation. Since this background methylation occurs more strongly within open chromatin [13], where the majority of transcription factors bind, any non-perfect control runs the risk of removing actual transcription factor binding signal. After normalisation with unfused Dam, binding profiles obtained by DamID only modestly correlate to ChIP signal and provide lowered spatial resolution due to the spread of methylation to several kb around binding sites [8, 10]. Indeed, the most successful use of DamID avoids these limitations entirely by studying nuclear lamin associated domains, which are much larger than the spatial resolution of DamID and whose heterochromatic organisation is negatively correlated with background Dam methylation [14, 15].

Dam methylates quite quickly; strong interactions with the DNA backbone lead it to remain bound afterwards, allowing it to processively methylate several GATCs at a stretch (including the reverse complement GATC) [16–18]. Coffin et al. [19] studied the structural basis of Dam processivity by mutating several basic residues of Dam that contact phosphates outside the active site [20]. These were found to change the balance between enzyme kinetics and DNA release, such that the rate of methylation became the slower, rate-limiting step. This has the effect of making the enzyme more likely to disassociate and float away instead of continuing on to methylate nearby sites.

We hypothesised that the features of these mutants—slower methylation rate, reduced DNA binding, or less processivity—could reduce the non-specific background methylation seen in DamID. Here we screened the effect of combinations of such mutations on DamID for the transcription factor Tcf7l2 and find that in general they greatly reduce the amount of non-specific methylation. The sparser methylation required altering the existing DamID-seq protocol to detect single methylation events instead of broader regions. The end result is DamID that gives a much cleaner signal for transcription factor binding, with sensitivity and spatial resolution comparable to levels seen with ChIP-seq.

## Results

### Mutant Dam protein maintains methylation sensitivity and increases specificity

**Fig. 1:**
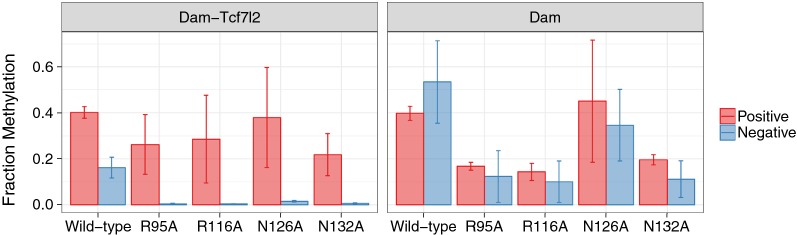
qPCR for Dam-Tcf7l2 methylation at control sites. Fraction of sequences methylated is estimated by qPCR at four positive Tcf7l2 ChIP-seq (red) regions compared to four DNase hypersensitive regions (blue) distal to any Tcf binding following expression of Dam or Dam-Tcf7l2 constructs for 8 (wild-type) or 24 h (mutants). Mean ± SD

DamID for Tcf7l2 binding was done in mouse embryonic stem cells (mESCs) with a single-integration Dox-inducible transgene expression cassette [21] carrying a Dam-Tcf7l2 or Dam only construct, with either wild-type Dam or one of the four mutations previously shown to reduce Dam binding and methylation rate (R95A, R116A, N126A, N132A). Initial screening was done by comparing the level of methylation at four positive sites; those bound according to Tcf7l2 ChIP-seq in mESCs, to four negative sites, which are at least 20 kb from any Tcf7l2 binding peaks yet still fall within accessible chromatin (DNase hypersensitive sites) (Fig. 1). After 8 h of Dox treatment, wild-type Dam-Tcf7l2 showed the expected enrichment of methylation at Tcf7l2 bound sites, along with substantial background methylation at control open chromatin sites that lack Tcf7l2 binding (2.5$\times$ increase from 0.16 to 0.40). Dam mutations reduced the overall amount of methylation; to maximise their signal, the Dox treatment was lengthened to 24 h (wild-type Dam-Tcf7l2 saturates in methylation by 24 h and is no longer enriched at Tcf7l2 bound sites). All four Dam-Tcf7l2 mutants showed a similar profile to each other after 24-h treatment: comparable methylation to wild-type Dam-Tcf7l2 at positive sites and negligible methylation at negative sites (fold enrichment: N126A 25x, N132A 36x, R116A 65x, R95A 65x). The same mutations in unfused Dam showed a marginal increase in methylation between these sites (1.3$\times$ to 1.8$\times$). Across both Dam-Tcf7l2 and Dam only constructs, the N126A variant retained the highest rate of total methylation (Dam-Tcf7l2: 0.38 positive, 0.015 negative; Dam: 0.45 positive, 0.35 negative) and R95A the lowest (Dam-Tcf7l2: 0.26 positive, 0.004 negative; Dam: 0.17 positive, 0.12 negative). All combinations (from pairwise to all) of these mutations, along with K139A/K140A, were also screened but showed undetectable levels of methylation, even at Tcf7l2-bound sites.

### Genome-wide DamID-seq protocol

**Fig. 2:**
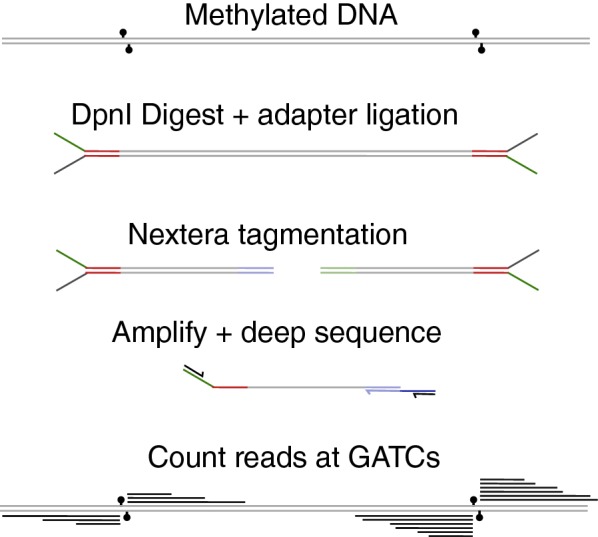
DamID-seq protocol. DamID-seq protocol. DpnI digestion produces blunt ends at all methylated sites, onto which a forked adapter containing an Illumina sequencing site is ligated. A second sequencing adapter is introduced randomly throughout the genome by a transposase (Nextera tagmentation). Fragments containing both adapters are amplified and directly sequenced. Following alignment, all reads originating at a GATC are summed to give an estimate of amount of methylation

To verify whether the improved specificity of mutant DamID holds for all Tcf7l2 binding sites, we sought to identify adenine methylation profiles genome-wide following expression of the wild-type, R95A, and N126A variants of Dam-Tcf7l2 and unfused Dam. Since the previously published DamID protocol only amplifies fragments ending with a methylated GATC at both ends (with no intervening unmethylated GATC) [7], we were concerned that it would miss any isolated methylated sites. This could mask any improvement caused by reduced noise or increased spatial resolution, as these would result in fewer methylated GATCs. We thus designed a protocol to detect genome-wide adenine methylation using Illumina NGS, which is summarized in Fig. 2. The initial steps are similar to other DamID protocols: a DpnI digestion produces blunt ends at all methylated sites, onto which an adapter is ligated. In our case, this is a forked adapter that contains an Illumina sequencing adapter for direct sequencing of methylated GATC sites. To produce fragments of appropriate size for Illumina sequencing, we introduce a second sequencing adapter through Nextera tagmentation, in which a transposase cuts and integrates sequences randomly throughout the genome. The resulting sequences are amplified using one primer specific to the ligated adapter and the other primer specific to one of the added Nextera sequencing adapters, such that every amplified fragment originally derives from an adenine-methylated site. We then perform paired-end Illumina NGS, obtaining 15-30 million reads per sample. Following alignment, any reads not originating from the restriction site in the middle of a GATC are removed. For wild-type Dam-Tcf7l2, and Dam only wild-type, N126A, and R95A, this retains the majority of reads (0.89 to 0.70). Due to their lower overall methylation, Dam-Tcf7l2 N126A and R95A retain fewer reads (0.29 and 0.20). An uninduced, negative control only retains 0.009 reads, which is close to the empirical background frequency of GATCs in the genome (0.0026). Finally, the use of paired-end sequencing shows that the majority of reads occur from a different tagmentation site (0.75 for Dam-Tcf7l2 N126A and R95A and 0.90 for unfused Dam and Dam-Tcf7l2 wild-type, consistent with the total amount of methylation sites) and hence correspond to an unique in-vivo methylated GATC and can be treated as independent measures.

### Mutant Dam protein reduces background methylation and improves spatial resolution

**Fig. 3:**
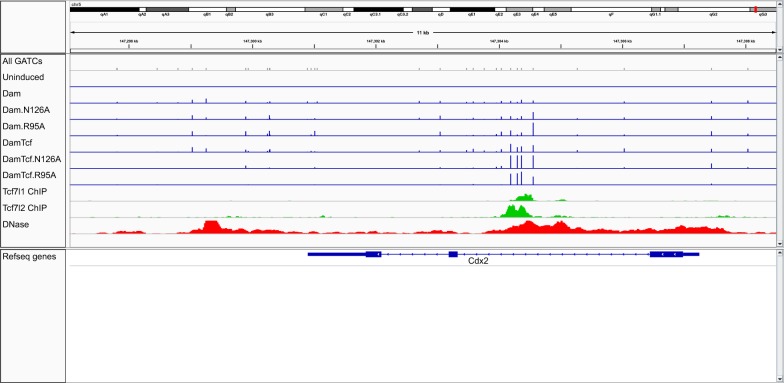
Dam-Tcf7l2 methylation at Cdx2. Methylation caused by Dam-Tcf7l2 and unfused Dam (wild type, N126A, and R95A) around a known Tcf7l2 bound enhancer in an intron of Cdx2. Top row shows position of all GATCs. Blue bars show the level of methylation at individual GATCs. Tcf7l1 and Tcf7l2 ChIP-seq signal are shown in green, DNase hypersensitivity in red. Scale is 0–50 read counts

**Fig. 4:**
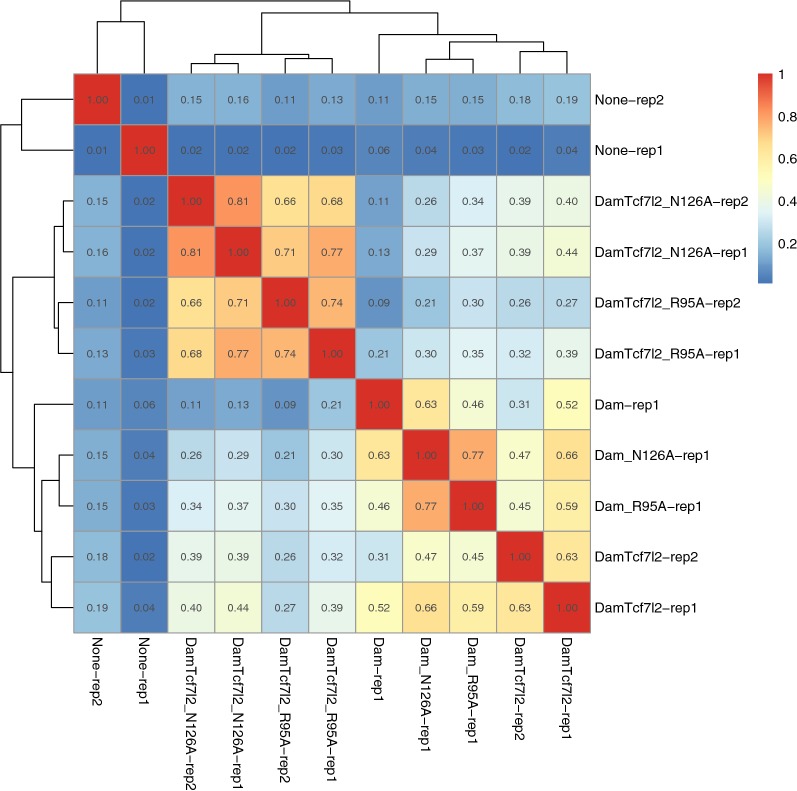
Correlation in Dam-Tcf7l2 methylation across the genome. Pearson correlation of raw read counts across all genomic GATCs. Rep 1 and Rep 2 are two replicates with high (15–30 million) and low (5–6 million) raw read count, respectively

Mapped reads from this DamID-seq protocol show clearly improved specificity of the R95A and N126A mutant versions of Dam-Tcf7l2 as compared to wild-type. The methylation pattern around a known Tcf7l2 bound enhancer located in an intron of Cdx2 is shown in Fig. 3. Both Dam-Tcf7l2 mutants (R95A and N126A) closely follow the Tcf7l2 ChIP-seq peaks in that region, with little methylation elsewhere. Wild-type Dam-Tcf7l2, on the other hand, shows less specificity for the ChIP-seq signal and a higher background, with a higher correlation to unfused Dam signal across the genome: 0.52–0.66, compared 0.13–0.37 for the Dam-Tcf7l2 mutants (Rep 1 in Fig. 4). A separate batch (rep 2 in Fig. 4) of Dam-Tcf7l2 wild-type and mutants with lower read depth and starting genomic material (wild type: 6 million, N126A: 1.5 million, R95A: 1 million GATC reads) cluster together with their respective constructs (wild type: 0.63, N126A: 0.81, R95A: 0.74). Additionally, the high similarity of N126 and R95A in both Dam-Tcf7l2 and unfused Dam provides a further measure of reproducibility, as both independently reduce the DNA binding and processivity of Dam.

**Fig. 5:**
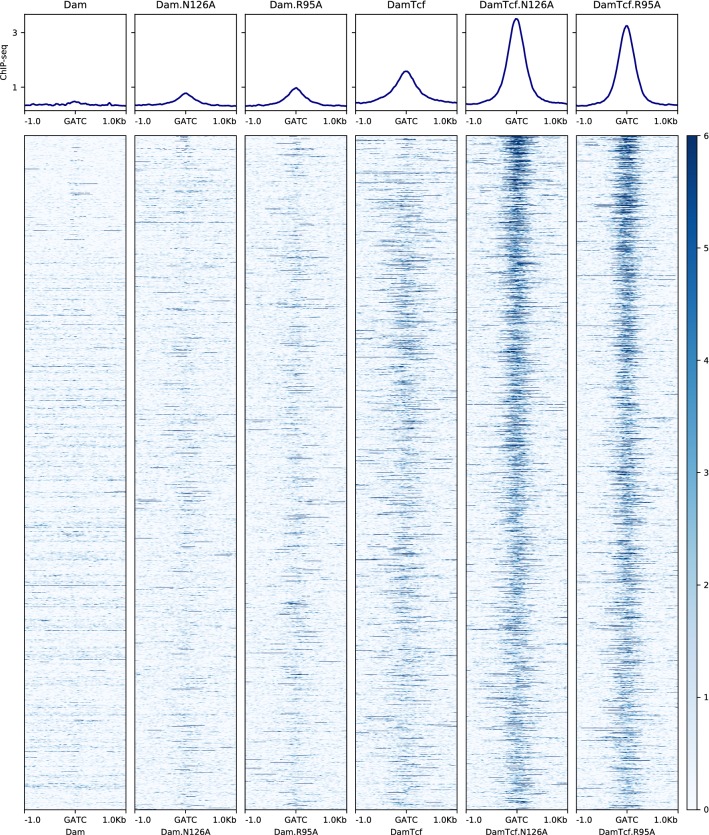
Tcf7l2 ChIP-seq signal around Dam-Tcf7l2 methylation. Tcf7l2 ChIP-seq signal (intensity in blue) around the top 6000 methylated sites in each sample. Each site is represented as a single line and is sorted from top to bottom by decreasing methylation levels

**Fig. 6:**
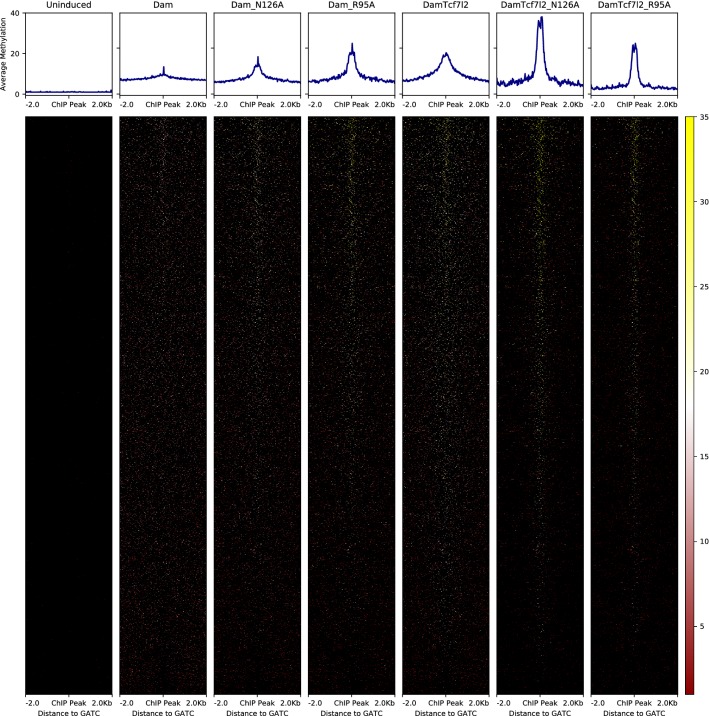
Decay in Dam-Tcf7l2 methylation from ChIP-seq peaks. Methylation at individual GATCs around Tcf7l2 ChIP-seq binding peaks. Each line is a single peak, with black denoting the background sequence (with no GATCs) and the red–white–yellow scale representing the amount of DamID reads at each GATC. To visualise methylation better each spot is a 20 bp non-overlapping window, so corresponds to a single GATC most of the time

Across the whole genome methylation by Dam-Tcf7l2, mutants colocalises much more strongly with Tcf7l2 ChIP-seq signal (Fig. 5). Normalising to unfused Dam controls doesn’t improve the colocalisation of wild-type or mutant Dam-Tcf7l2 signal with Tcf7l2 ChIP-seq, instead reducing it in all constructs (Additional file 1: Fig. S1). Similarly, the two Dam-Tcf7l2 mutants also exhibit higher sensitivity for ChIP-seq signal, with far less methylation away from binding sites (Fig. 6). Dam-Tcf7l2 mutant methylation decays to half very quickly from the midpoint of the ChIP-seq peak, at 120 bp (R95A) and 160 bp (N126A), and falls to a rate of 2% of peak methylation at 1 kb away. Wild-type Dam-Tcf7l2 reaches half methylation at 580 bp away from the midpoint, and at 1 kb still methylates on average 25% as much. Since this measure is specific to the ChIP-seq sites and could be confounded by higher background methylation or difference in what actual signal it detects, we also checked whether this pattern appears in the autocorrelation of methylation signal—how similar it is between nearby segments (averaged across 100 bp bins) and hence how fast the signal varies. This supports an increase in spatial resolution with the mutants, with wild-type Dam-Tcf7l2 signal still correlated across 1–2 kb, by which point the mutant signal is uncorrelated (Additional file 2: Fig. S2). Lastly, the reduced background methylation and increased spatial resolution of the mutants puts it in the range of the distribution of GATCs throughout the genome. This results in Tcf7l2 bound sites that are captured by only one methylated site and hence would be missed with the classic DamID protocol (Additional files 3 and 4: Fig. S3 and Fig. S4).

## Discussion

Here we show that four mutants of Dam (R95A, R116A, N126A, and N132A) each reduce the noise seen in DamID for the transcription factor Tcf7l2 substantially, and for two of these (R95A and N126A) we confirm that this is the case across the whole genome, resulting in less background methylation and higher spatial resolution. We strongly suspect that these conclusions will also apply to the other two mutants.

We are not sure precisely what causes the background methylation observed with wild-type Dam, and hence, why these mutants show an increased signal-to-noise ratio. Based on the observations in [19] of such mutations, it could be a combination of reduced methylation rate leading to only longer-lived interactions being recorded, lower processivity preventing spreading methylation, or reduced DNA binding preventing it from dragging its linked transcription factor to a new location. The observation that unfused Dam mutants closely resemble the wild-type Dam-Tcf7l2 favours the last of these: that wild-type Dam binds DNA strongly enough to drag Tcf7l2 to locations that Dam normally prefers. If the improved signal was instead due to disrupted processivity, then the correlation between wild-type Dam and Dam-Tcf7l2 should be stronger than that between mutant Dam and wild-type Dam-Tcf7l2. Alternatively, if the cause was a reduced methylation rate only capturing longer-lived interactions, then one would expect the mutant Dam only samples to show less total methylation than the corresponding Dam-Tcf7l2—the opposite was observed.

A caveat to our results is that these Dam constructs were expressed from a dox-inducible promoter at high level, in contrast to the recommended method of using low expression from a leaky uninduced promoter. It is possible that there exists a lower concentration and duration of Dam-Tcf7l2 with similar signal-to-noise properties as the N126A and R95A variants. During out initial test of wild type Dam-Tcf7l2, however, we found no concentration or duration of dox exposure that further improved the enrichment at Tcf7l2 bound sites (by qPCR) nor did the uninduced promoter provide detectable signal (these observations may be specific to the quick replication of mESCs diluting away methylation that is produced too slowly). Furthermore, previous studies all show low spatial resolution and high correlation between unfused Dam and fusions with transcription factors despite attempts to maintain low levels of expression.

Out of these, the most comparable is a recent DamID experiment by Cheetham et al. [10] profiling Oct4 binding in mESCs, due to the explicit comparison to ChIP-seq and similarly focal DNA binding of Tcf7l2 and Oct4 with ChIP-seq peaks of $\sim$ 100 bp. Despite maintaining very low expression of Dam-Oct4 fusion through translation reinitiation, a comparison with Oct4 ChIP-seq shows methylation at many disparate sites and a low spatial resolution similar to what we observe for Dam-Tcf7l2 wild-type (50% decay at >  500 bp). While a portion of these may be true Oct4 binding events, the high specificity of ChIP-seq for transcription factor binding, combined with the high correlation (median of 0.77) to unfused Dam, indicates that this is mostly driven by Dam-specific effects. This matches our observations for Tcf7l2 fused to wild-type Dam, which is more strongly correlated with unfused Dams than the N126A or R95A Dam-Tcf7l2. Thus, it seems unlikely that the increase in signal-to-noise seen with the Dam mutants is achievable through further optimisation of Dam fusion expression. More generally, the strong DNA binding and processivity of wild type Dam [16–18] indicates that for any protein with similar or weaker DNA affinity, fusing it to Dam will result in off-target methylation regardless of the level of total methylation. Very low expression also adds an additional source of cell-to-cell variability due to stochastic fluctuations inherent with few mRNAs.

The spreading methylation of wild type Dam spans across multiple GATCs. The more localised methylation by mutant Dam, however, makes the frequency of GATCs the new limit for spatial resolution. We addressed this by developing a DamID-seq protocol that captures individual methylated sites, rather than reading out the correlation between adjacent pairs, which increases how frequently methylation is sampled across the genome; several Tcf7l2 binding sites were detected by only a single GATC. Additionally, this protocol reduces the number of steps required by using the initial ligated adapter directly for sequencing, instead of separating amplification of methylated fragments from later sequencing library preparation (as in [7]), and produces a more interpretable output of read count at each GATC instead of being smeared out into a peak. Further increasing the frequency with which binding can be detected could be achieved by combining these Dam mutants with K9A, which allows Dam to methylate at sequences other than GATC and detecting the resulting methylation by immunoprecipitation [22, 23].

The recommended method for dealing with background activity is to express an unfused Dam control and hope that it recapitulates the off-target methylation of the fusion construct. Interestingly, when we tried this we instead got a decrease in signal with respect to Tcf7l2 ChIP-seq. Since both background Dam methylation and transcription factor binding tend to occur within open chromatin regions, the unfused Dam control is already partially predictive of binding sites. Confounding factors, such as differences in background methylation rates between unfused Dam and Dam-Tcf7l2 due to higher diffusion of the small unfused Dam, would result in normalisation creating false negatives.

A previous paper has proposed the Dam mutant L122A to increase the signal to noise of DamID. They however report a higher correlation ($\sim$ 0.7) between unfused Dam and the Dam transcription factor fusion compared to ours (Fig. 4) and provide no evidence for the claim of increased signal-to-noise of the L122A mutant [12]. Additionally, this mutant was reported to show a preference for methylating already hemimethylated sites [20, 24]. While of interest as a possible way to maintain Dam methylation through DNA replication, preferential propagation of existing methylation throughout cell division would abolish independence between individual methylation events, confounding any statistical inference.

In this study, we focused on a specific transcription factor, Tcf7l2, and showed that mutations in Dam improved detection of its binding to DNA. Owing to the absence of any unique features of Tcf7l2—it neither binds particularly strongly nor has easy to predict binding—we would expect that these benefits should apply generally to other transcription factors. Since the correlation between unfused Dam mutants and wild-type Dam-Tcf7l2 suggests that off-target effects are due to strong DNA-binding of Dam, rather than processivity or kinetics, DamID generally would be most reliable for strongly binding proteins, such as CTCF, pioneer factors, or Cas9, while mutant Dam would have the most benefit for more transiently binding proteins.

With the growing appreciation of cellular heterogeneity, it is of interest to study transcription factor binding in finer resolution than the bulk cell cultures or tissues that are required by ChIP-seq. DamID provides unique benefits for measuring protein-DNA interactions in such situations, as the construct can be expressed in response to certain perturbations or in specific cell types—including within a whole organism—and easily isolated later due to the persistence of adenine methylation throughout further experimental processing. Due to the presence of artefacts in ChIP-seq, DamID is also of use in independently verifying binding sites, particularly those lacking a clear motif to explain binding. Since the noisiness of DamID has been a constant barrier to applying it more broadly, we hope that these improvements to its specificity and sensitivity for transcription factor binding will aid in the development of such experiments.

## Materials and methods

### Embryonic stem cell culture

All experiments were done in 129P2/OlaHsd mouse embryonic stem cells (mESC), which were cultured according to previously published protocols [25]. mESCs were maintained on gelatin-coated plates feeder-free in mESC media composed of Knockout DMEM (Life Technologies) supplemented with 15% defined foetal bovine serum (FBS) (HyClone), 0.1 mM nonessential amino acids (NEAA) (Life Technologies), Glutamax (GM) (Life Technologies), 0.55 mM 2 -mercaptoethanol (b -ME) (Sigma), 1X E SGRO LIF (Millipore), 5 nM GSK-3 inhibitor XV and 500 nM UO126. Cells were regularly tested for mycoplasma.

### Dam Tcf7l2 fusion constructs

Constructs were made by fusing Dam to the N-terminus of Tcf7l2 with a short flexible linker. Dam-Tcf7l2 and unfused Dam containing plasmids were integrated at one copy into mouse embryonic stem cells using a previously established p2Lox system [21]. This puts the Dam constructs under control of a tet-responsive promoter, along with integrating a neomycin resistance gene that is selected for by culturing the cells in G418 (300 $\upmu$g/mL) for 1 week.

Mutant versions of Dam and Dam-Tcf7l2 were created by electroporating in plasmids coding for Cas9 and a sgRNA targeting the middle of Dam, along with a template oligo containing the Dam sequence with each possible combination of R95A, R116A, N126A, N132A, K139A/K140A. This template also contains several noncoding mutations that disrupt the sgRNA site, such that the template gets integrated by homologous recombination due to a CRISPR/Cas9 induced cut within the Dam coding sequence, but doesn’t get itself cut after. Individual clones were chosen by flow cytometric sorting individual cells into a few 96-well plates. After growing for a week, a portion of cells were taken and the relevant portion of Dam amplified with primers containing a unique combination of barcodes for each well. This was sequenced on an Illumina Miseq sequencer (paired end 150 + 150 bp) to identify which well contained which mutation.

Dam constructs were expressed by the addition of doxycycline (500 ng/ul). Wild-type constructs were expressed for 8 h, as longer expression resulted in saturating methylation and no signal. All mutant constructs showed lower overall methylation rates and were expressed for 24 h. Beyond this, there is no further increase in methylation, presumably due to it reaching a steady state with dilution during cell division.

Genomic DNA was extracted using the Purlink kit (Invitrogen #K182001). Methylated sites were digested by DpnI (20 ul reaction, 10U DpnI, 2 ul Cutsmart buffer, 500 ng genomic DNA). Similarly, unmethylated sites were digested by DpnII (20 ul reaction, 25U DpnII, 2 ul DpnII buffer, 500 ng genomic DNA).

### Locus-specific DamID qPCR

Methylation was measured at specific sites by qPCR (20 ul from Syber master mix with 1ul restriction digest) with primers flanking a GATC following a DpnI or DpnII digest. The fraction methylated was calculated from the difference in cycle counts following DpnI (c_I_) and DpnII (c_II_) digestion: $\frac{1}{1 + 2^{c_{II}-c_I}}$. Positive controls were chosen from sites containing a GATC within a clear Tcf7l2 ChIP-seq peak in both embryonic stem and intestinal endoderm cells. This was compared to four similarly chosen negative controls that were at least 20 kb away from any Tcf binding event in either cell type yet still within an open chromatin (DNase hypersensitive) region. The specific locations and primers used for these sites are in Table 1.

**Table 1:** Location of and primers for sites that are positive or negative (yet still in open chromatin) for Tcf7l2 binding

Chr	Start	End	Forward primer	Reverse primer
*Positive controls*				
14	49155664	49155815	AAACCACTCTCCCCCAAAGC	TTTGAAGTTCCGGAGCGGTT
15	30383190	30383333	CTTAAAAGCAGGCTCCCTCGC	TCCACACTTCAAAAGGAGAGAAAG
4	129251719	129251895	ATTTCAAACAAACTCCCCGCTG	TGGAATTAGTTTGGGGCTCTGAT
5	74953049	74953184	AAGTGACCCTTTGTTCTCTGTC	CAAAGAATGGGCCGGGATG
*Negative controls*				
14	111679747	111681304	ACAGCTTCACTTCCTTGCCA	TTTGAATGAGGGAAGTCAGCT
7	10494816	10495738	GCCCTTAGAACCGCTCCTTT	TCCAGATCGTGTGCAAGACC
1	99772205	99772613	ACTATTGGTGGAGCTGTGCG	TGCTTGCCTTTCTTGCTTGC
3	111370868	111371261	AAGCAGCAAGAGGGAACACT	TGCATGCCACAGAATACTTTTAA

### Genome-wide DamID-seq

The genome-wide DamID-seq protocol is shown in Fig. 2. Detecting methylated GATCs throughout the genome was done by ligating on a sequencing adapter to any DpnI created cuts. A second adapter close to the ligated one was added with a nextera library prep kit, which fragments and inserts adapters randomly throughout the genome with a transposase. Fragments with one ligated adapter and one nextera adapter are amplified up and sequenced directly. Adapters for ligation were made by mixing the following two oligos at 50 uM, heating up to 95 $^\circ$C, then slowly cooling at 1 $^\circ$C per minute to anneal the two strands.

1. AATGATACGGCGACCACCGAGATCTACACTCTTTCCCTACACGACGCTCTTCCGATCT
2. AGATCGGAAGAGCGGTTCAGCAGGAATGCCGAGACCG

This creates a forked sequence with one blunt end (5$^\prime$ end of #1 with 3$^\prime$ end of #2). These were ligated onto methylated GATCs in a 20 ul T4 ligation (5U T4, 2 uM annealed adapters, T4 ligase buffer) with 10 ul of DpnI digested genomic DNA (25 ng/ul) at 16 $^\circ$C overnight, followed by inactivation at 65 $^\circ$C for 10 min. This ligation mixture was used directly as input for the nextera tagmentation, which was done according to the manufacturers instruction with the following changes:

1. Only one barcoded adapter (i7) is included for amplification instead of both. Due to suppression PCR only fragments with both a ligated and nextera adapter are amplified.
2. 9 cycles are used for amplification instead of 5 (since fewer fragment are being amplified).
3. A higher AMPure bead concentration is used (1.6$\times$ instead 0.6$\times$) to ensure we capture the smaller size distribution of our fragments, which stems from the transposase’s preference for DNA ends.

The resulting fragments are directly sequenced on an Illumina Nextseq sequencer with midoutput 150 bp kit (110 bp read one, 48 bp read two, and 8 bp index 1). Raw reads were aligned to the mm10 genome with BWA (mem algorithm with default parameters) [26]. Any reads not originating from the midpoint of a GATC (cut site of DpnI) at read one were presumed to be the result of non-specific ligation onto broken DNA ends and removed. The remainder were summed to give a read count per GATC.

## Additional files

**Additional file 1: Fig. S1.** Tcf7l2 ChIP-seq signal around normalised Dam-Tcf7l2 methylation. Tcf7l2 ChIP-seq signal (intensity in blue) around the top 6000 Dam-Tcf methylated sites after normalising with the corresponding unfused Dam control. Each site is represented as a single line, and are sorted from top to bottom by decreasing normalised methylation levels.

**Additional file 2: Fig. S2.** Autocorrelation in genomic Dam-Tcf7l2 methylation. Autocorrelation (Pearson) of methylation counts across the genome (averaged in 100 bp bins) in Dam-Tcf7l2 wildtype, N126A, and R95A samples.

**Additional file 3: Fig. S3.** Tcf7l2 peak captured by single GATC (1). Example of isolated GATC that picks up a Tcf7l2 binding peak. Top row shows position of all GATCs. Blue bars show the level of methylation at individual GATCs. Tcf7l1 and Tcf7l2 ChIP-seq signal are shown in green, DNase hypersensitivity in red. Scale is 0–50 read counts.

**Additional file 4: Fig. S4.** Tcf7l2 peak captured by single GATC (2). Another example of isolated GATC that picks up a Tcf7l2 binding peak (at left ChIP-seq peak). Top row shows position of all GATCs. Blue bars show the level of methylation at individual GATCs. Tcf7l1 and Tcf7l2 ChIP-seq signal are shown in green, DNase hypersensitivity in red. Scale is 0–50 read counts.

## Data Availability

The data presented in this article has been deposited in NCBI’s Gene Expression Omnibus and is accessible through accession number GSE125447(https://www.ncbi.nlm.nih.gov/geo/query/acc.cgi?acc=GSE125447). The raw sequencing data can be accessed via NCBI’s Short Read Archive (SRA) through accession number SRP181175.
